# Increased Lipid Peroxidation May Be Linked to Ferritin Levels Elevation in Adult-Onset Still’s Disease

**DOI:** 10.3390/biomedicines9111508

**Published:** 2021-10-20

**Authors:** Po-Ku Chen, Kai-Jieh Yeo, Po-Hao Huang, Shih-Hsin Chang, Ching-Kun Chang, Joung-Liang Lan, Der-Yuan Chen

**Affiliations:** 1 Rheumatology and Immunology Center, China Medical University Hospital, Taichung 404, Taiwan; pago99999@gmail.com (P.-K.C.); D30870@mail.cmuh.org.tw (K.-J.Y.); u402082@gmail.com (P.-H.H.); sherry61976@hotmail.com (S.-H.C.); kun80445@gmail.com (C.-K.C.); jounglancmuh@gmail.com (J.-L.L.); 2 College of Medicine, China Medical University, Taichung 404, Taiwan; 3 Translational Medicine Laboratory, China Medical University Hospital, Taichung 404, Taiwan; 4 Translational Medicine and Rong Hsing Research Center for Translational Medicine, National Chung Hsing University, Taichung 402, Taiwan; 5 Rheumatic Diseases Research Center, China Medical University Hospital, Taichung 404, Taiwan

**Keywords:** lipid peroxidation (LPO), LPO inducer, LPO-related metabolites, ferritin, adult-onset Still’s disease (AOSD)

## Abstract

Lipid peroxidation (LPO) and hyper-ferritinemia are involved in inflammatory responses. Although hyper-ferritinemia is a characteristic of AOSD, its link to LPO remains unclear. We investigated the association between LPO and ferritin expression, and evaluated the relationship between LPO-related metabolites and inflammatory parameters. Mean fluorescence intensity (MFI) of LPO (C11-Biodipy^581/591^)-expressing PBMCs/monocytes in AOSD patients and healthy control (HC) subjects was determined by flow-cytometry analysis. Expression of ferritin and cytokines on PBMCs/macrophages was examined by immunoblotting. Plasma levels of LPO-related metabolites and cytokines were determined by ELISA and the MULTIPLEX platform, respectively. LPO MFI on PBMCs/monocytes were significantly higher in patients (median 4456 and 9091, respectively) compared with HC (1900, *p* < 0.05, and 4551, *p* < 0.01, respectively). Patients had higher ferritin expression on PBMCs (mean fold, 1.02) than HC (0.55, *p* < 0.05). Their ferritin expression levels on PBMCs stimulated with LPO inducers erastin or RSL3 (2.47 or 1.61, respectively) were higher than HC (0.84, *p* < 0.05, or 0.74, *p* < 0.01). Ferritin expression on erastin-treated/IL-1β-treated macrophages from patients were higher than those from HC (*p* < 0.001). The elevated levels of LPO-related metabolites, including malondialdehyde and 4-hydroxyalkenals, were positively correlated with disease activity scores, suggesting LPO involvement in AOSD pathogenesis. Increased ferritin expression on PBMCs/macrophages stimulated with LPO inducers indicates a link between LPO and elevated ferritin.

## 1. Introduction

Adult-onset Still’s disease (AOSD) is characterized by fever, rash, arthralgia or arthritis, liver dysfunction, multi-systemic involvement, and increased acute-phase reactants, including hyper-ferritinemia [1,2]. AOSD is a rare but important cause of fever of unknown origin [3]. The reported incidence rates of AOSD were 0.16, 0.22, and 0.4 per 100,000 persons in west France [4], Japan [5], and northern Norway [6], respectively. Aberrant immune system activation may lead to increased pro-inflammatory cytokines, including interleukin (IL)-1β, IL-6, IL-18, and tumor necrosis factor (TNF)-α [7,8,9,10]. Therefore, biologics targeting IL-1, IL-6, or IL-18 have been proven effective in treating AOSD [10,11,12,13,14,15]. Given its clinical phenotypes and the absence of detectable autoantibodies, AOSD is considered an autoinflammatory disease (AID) [16] with inflammasomes’ dysregulation [17,18]. We have recently revealed the pathogenic roles of NOD, LRR, and pyrin domain-containing protein 3 (NLRP3) inflammasome signaling in AOSD [19].

Hyper-ferritinemia, a significant feature of several autoimmune [20] and autoinflammatory diseases [21], may not only reflect an acute-phase response but may play a critical role in the inflammation [22] and immunomodulation in these diseases [23]. Ferritin is a major intracellular iron storage protein composed of 24 heavy (H)- and light (L)-chain subunits, and the ratio between these two subunits may differ depending on tissue types and physiologic statuses [24]. Levi et al. demonstrated that L-chain apoferritin (FTL) has a higher capacity than H-chain apoferritin (FTH) to induce iron-core nucleation, whereas H-chain ferritin is superior in promoting Fe^++^ oxidation [25]. Beyond its iron storage role, ferritin participates in the pathogenesis of inflammation [26] and may stimulate and amplify the inflammatory processes [27]. FTH displays both immunomodulatory and pro-inflammatory functions and induces the expression of different inflammatory mediators, such as IL-1β [28]. Exogenous FTH stimulation may regulate NLRP3 inflammasome signaling and IL-1β production on macrophages [29]. Serum ferritin levels were found to be correlated with disease activity and macrophage activation [21,30]. Besides, Thomas et al. have shown that ferritin is a good source of iron for catalysis of lipid peroxidation (LPO) [31]. Daily supplementation with iron may increase LPO in young women [32], and an excess of free Fe^++^ can promote the production of radical oxygen species (ROS) [33]. Therefore, cellular iron metabolism, including in iron import, storage, utilization, and export, should be strictly regulated [34].

Oxidative stress is defined as the imbalance between the production of reactive oxygen species (ROS) and antioxidants, and excess ROS production may induce LPO and cause cell death through apoptosis, autophagy, and ferroptosis [35]. Ferroptosis is an iron-dependent form of regulated cell death, distinct from apoptosis, autophagy, and other forms of cell death [36]. Cysteine-glutamate transporter System Xc (xCT), a heterodimer transmembrane protein, enhances cysteine uptake, generates glutathione biosynthesis, and may act as a cofactor for glutathione peroxidase 4 (GPX4) [37]. GPX4 can combine with GSH to co-localize lipid peroxides in cells and then prevent ferroptosis. A class of ferroptosis inducers, such as erastin, reduces the concentration of intracellular cystine by inhibiting xCT, while RSL3 induces ferroptosis by inhibiting GPX4 [38]. Increasing evidence indicates that ferroptosis is associated with reduced detoxification of lipid peroxides by GPX4, and the peroxidation of polyunsaturated fatty acids (PUFAs) was considered a key driver of ferroptosis [36]. Besides, malondialdehyde (MDA) has been widely used as a convenient biomarker for lipid peroxidation because of its facile reaction with thiobarbituric acid (TBA) [39] and mediators of inflammatory responses [40]. The 4-hydroxyalkenals (4-HNE), an aldehyde product of membrane lipid peroxidation, is nowadays considered as a major bioactive marker of lipid peroxidation because it is produced in relatively large amounts [39,41]. However, there is not any data regarding LPO stress or LPO-related metabolites in AOSD.

This pilot study investigates the differences in LPO levels in circulating monocytes and peripheral blood mononuclear cells (PBMCs) between AOSD patients and healthy control (HC) subjects. We also examine the effect of LPO inducers, including erastin and RSL3, on the protein expression levels of FTH, FTL, pro-IL-1β, IL-1β, and IL-18 on PBMCs or macrophages derived from AOSD patients and HC subjects, respectively. Besides, we compare the plasma levels of MDA and 4-HNE, the LPO products, between AOSD patients and HC subjects and then evaluate the correlation between the levels of LPO-related metabolites and disease activity scores or inflammatory parameters in AOSD patients.

## 2. Materials and Methods

### 2.1. Patients and Study Design

In this prospective study, 25 AOSD patients who fulfilled the Yamaguchi criteria [42] were enrolled consequently. Systemic disease activity was assessed with a modified Pouchot score [43]. This systemic activity score (range 0–12) assigns one point to each of 12 manifestations: fever, evanescent rash, sore throat, arthralgia or arthritis, myalgia, pleuritis, pericarditis, pneumonitis, lymphadenopathy, hepatomegaly or abnormal liver function, elevated leukocyte count ≥15,000/mm^3^, and serum ferritin levels >3000 µg/L. Active AOSD was defined as systemic activity scores of at least 4 [44]. All patients were treated with corticosteroids with or without the non-steroidal anti-inflammatory drugs (NSAIDs) at an active status. Besides, the disease-modifying anti-rheumatic drugs (DMARDs) prescribed included methotrexate (*n* = 16), hydroxychloroquine (*n* = 18), azathioprine (*n* = 4), cyclosporine (*n* = 7), and tocilizumab, an IL-6 receptor inhibitor (*n* = 4). A higher proportion of active AOSD patients received cyclosporine (5, 35.7%) and tocilizumab (3, 21.4%) compared with inactive patients (2, 18.2% and 1, 9.1%, respectively), but no statistical significance was found. Fourteen healthy volunteers who had no rheumatic disease were enrolled as control subjects. This study was approved by the Institutional Review Board of the Chinese Medicine University hospital (CMUH109-REC1-108, Date of approval: 8 September 2020), and informed consent was obtained from each participant according to the Declaration of Helsinki.

### 2.2. Quantitation of C11-BODIPY^581/591^ Levels Using Flow Cytometry Analysis

PBMCs were immediately isolated from whole blood using the Ficoll-PaqueTM PLUS (GE Healthcare Biosciences, Chicago, IL, USA) density gradient centrifugation, and then reacted with 500 μL of RBC Lysis Solution (BD Biosciences, San Jose, CA, USA) for 5 min to lyse red blood cells. PBMCs (2 × 10^5^ cells) were stained with 2 μM of C11-BODIPY^581/591^ (Life Technologies-Invitrogen, Carlsbad, CA, USA), a lipophilic ROS-sensitive probe indicating LPO (Thermo Fisher Scientific, Inc., Waltham, MA, USA), for 30 min and then washed with phosphate-buffered saline (PBS). The cells were stained with Allophycocyanin (APC)-conjugated anti-CD14 monoclonal antibody (mAb) (BD Biosciences, San Jose, CA, USA), according to the manufacturer’s protocol and the described technique [45]. Fluorescent antibodies, mouse IgG2ακ-APC (BD Biosciences, San Jose, CA, USA), were used as isotype controls. The cells were centrifuged at 1800 rpm for 5 min, and then the supernatant was discarded. After being resuspended with 200 μL of PBS, they were analyzed by a BD FACSCelesta™ Flow Cytometer (BD Biosciences, San Jose, CA, USA). The PBMCs were gated based on the dot-plots of forward scatter (FSC) and side scatter (SSC), and then monocytes were included and analyses of CD14^+^ cells were performed. To determine C11-BODIPY^581/591^ expressions, we set the gating mode using unstained samples of PBMCs by FSC and SSC of light as background fluorescence. For analyzing the percentage of C11-BODIPY^581/591^ on CD14^+^ cells, the gate was set to determine CD14^+^ cells, and C11-BODIPY^581/591^ levels were calculated on the cell population. Data were expressed as the mean fluorescence intensity (MFI) or the percentages of C11-BODIPY^581/591^ expressions in circulating PBMCs or CD14^+^ cells (monocytes).

### 2.3. In Vitro Cell Studies

The PBMCs derived from active AOSD patients and HC subjects were rested for 1 h in the completed culture RPMI 1640 medium (Thermo Fisher Scientific, Inc., Waltham, MA, USA) with 10 mM of HEPES at 37 °C in an incubator, and then placed in culture RPMI 1460 medium with 10 ng/mL of granulocyte macrophage-colony stimulating factor (GM-CSF) (R&D Systems, Minneapolis, MN, USA) for 7 days to induce the macrophage differentiation [46]. To examine the effect of LPO inducers, including erastin and RSL3, on the expression of ferritin, FTL, FTH, IL-1β, and IL-18 on the PBMCs or macrophage isolated from AOSD patients and HC subjects, cells were incubated with different treatments for 24 h. 

The proteins were extracted with Cell lysis buffer (Cell Signaling Technology, Danvers, MA, USA) supplemented with complete EDTA-free protease inhibitor cocktail (Roche) on ice and then stored at −80 °C until use. The concentration of proteins was determined by the BCA assay kit (Thermo Fisher Scientific, Inc., Waltham, MA, USA). The proteins (10 μg) were separated with 12.5% SDS-PAGE and then transferred on 0.45 μm PVDF membranes (PerkinElmer, Foster City, CA, USA). The membranes were blocked with 5% skimmed milk in PBS containing 0.1% Tween-20 (Bionovas, Inc., Washington, DC, USA) at room temperature for 30 min. Immunoblots were performed using specific primary antibodies against anti-ferritin, anti-xCT, anti-GPx4, anti-FTL and anti-FTH antibodies (Abcam, Cambridge, MA, USA), anti-pro-IL-1β and anti-IL-1β antibodies (NOVUS, Littleton, CO, USA), anti-IL-18 and anti-CD68 antibodies (Santa Cruz Biotechnology, Dallas, Texas, USA), and anti-GAPDH antibody (Elabscience, Houston, TX, USA) at 4 °C overnight. Immunoreactive bands were visualized using an enhanced chemiluminescence detection system (Millipore, Billerica, MA, USA), and band intensity was determined by Image J software. The protein expression levels of ferritin, FTL and FTH, CD68, pro-IL-1β, IL-1β, and IL-18 were normalized to GAPDH.

### 2.4. Investigation of Cell Viability by WST-1 Assay

PBMCs from patients and HC subjects were seeded onto 96-well plates. Cells were incubated with a ferroptosis inducer, including 20 μM of erastin (Sigma-Aldrich, St. Louis, MO, USA) or 10 μM of RSL-3 (Sigma-Aldrich, St. Louis, MO, USA) for 24 h at 37 °C. The cell viability of PBMCs was determined by the WST-1 assay as a cell proliferation reagent (Merck Millipore, Temecula, CA, USA). After incubating the WST-1 reagent for 2 h, absorbance was monitored at 440 nm using an ELISA reader.

### 2.5. Determination of Plasma Levels of Malondialdehyde (MDA) and 4-Hydroxynonenal (4-HNE)

Whole blood was collected in blood collection EDTA tubes (BD Biosciences, San Jose, CA, USA), and the obtained plasma samples were centrifuged at 2000 rpm for 10 min. Plasma samples were stored at −80 °C until use. Plasma MDA levels were determined by a commercial MDA kit by quantification of thiobarbituric acid reactive substance (TBARS) (Biovision, Milpitas, CA, USA) [47], and 4-HNE-BSA levels were measured by an ELISA kit (Abcam, Cambridge, MA, USA) according to the manufacturer’s instructions.

### 2.6. Determination of Plasma Levels of Cytokine Profiles 

To avoid the potential variability in cytokines’ quantification across the platform, plasma levels of IL-1β, IL-1 receptor antagonist (IL-1Ra), IL-6, IL-18, and TNF-α were determined by magnetic multiplex using a MULLIPLEX^®^ Human Cytokine/Chemokine/Growth Factor Panel A (Cat# HCYTOMAG-60K-16) according to the manufacturer’s instructions (Milliplex MAP kits, EMD Millipore, Billerica, MA, USA).

### 2.7. Statistical Analysis

Results are presented as the mean ± standard deviation (SD) or median (interquartile range). The Bonferroni post-test and Kruskal–Wallis test were used for comparisons between groups. When the tests showed a significant difference, the exact *p*-value was determined using the Mann–Whitney U test. The correlation coefficient was calculated using the nonparametric Spearman’s rank correlation test. A *p*-value < 0.05 was considered statistically significant.

## 3. Results

### 3.1. Clinical Characteristics of AOSD Patients

Among the 25 AOSD patients, spiking fever (≥39 °C), rash, arthralgia or arthritis, sore throat, liver dysfunction, and lymphadenopathy were noted in 23 (92.0%), 21 (84.0%), 18 (72.0%), 16 (64.0%), 9 (36.0%), and 7 (28.0%) patients respectively, in the initial active status. There were no significant differences in the age at study entry or the proportion of females between AOSD patients (mean age ± SD, 38.7 ± 9.0 years, and 80.0%, respectively) and healthy subjects (38.5 ± 3.7 years and 78.6%, respectively).

### 3.2. The Levels of C11-BODIPY^581/591^ Fluorescence Intensity on PBMCs and CD14^+^ Cells in AOSD Patients and Healthy Control (HC) Subjects

**Figure 1:**
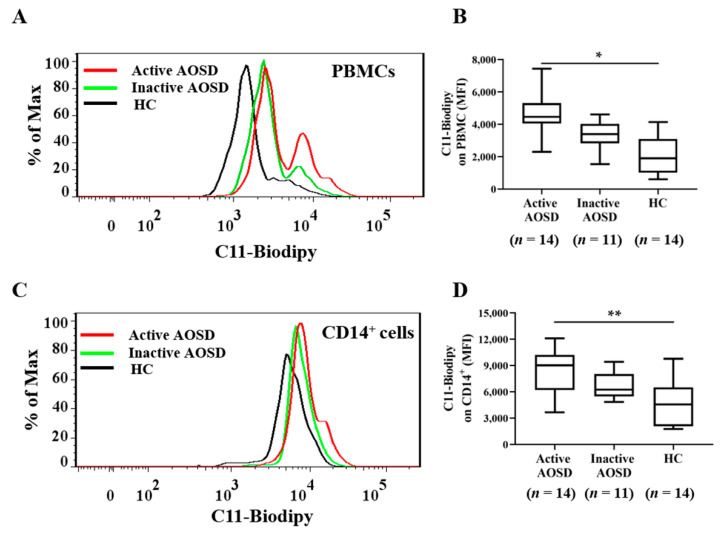

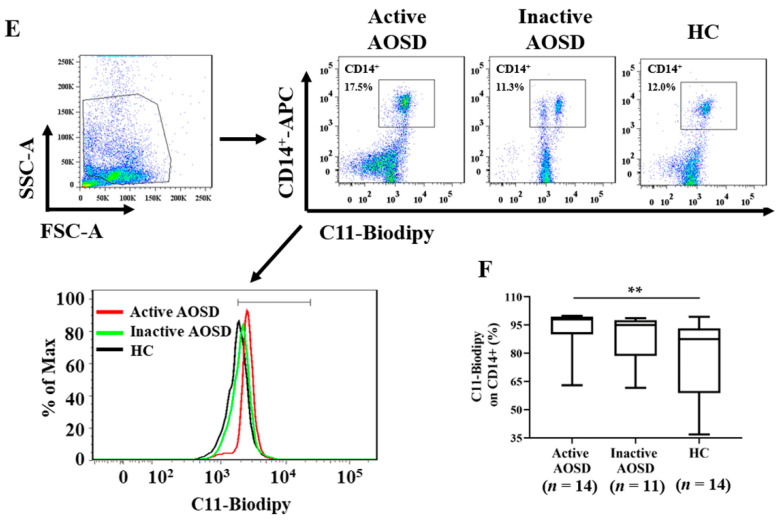
The C11-BODIPY^581/591^ fluorescence intensity on PBMCs and CD14^+^ cells from adult-onset Still’s disease (AOSD) patients and healthy control (HC) subjects. The representative histograms of the C11-BODIPY^581/591^ fluorescence intensity on circulating PBMC-gated cells (**A**) or CD14^+^-gated cells (**C**) obtained from one active AOSD patient, one inactive AOSD patient, and one HC subject. Comparisons of the mean fluorescence intensity (MFI) of C11-BODIPY^581/591^ on PBMC-gated cells (**B**) or CD14^+^-gated cells (**D**) among active AOSD patients, inactive AOSD patients, and HC subjects. (**E**) Dot-plots of C11-BODIPY^581/591^ level on circulating allophycocyanin (APC)-CD14^+^ cells from one of the different groups. After gating on monocytes (CD14+ cells), the percentages of positive C11-BODIPY^581/591^ were calculated. (**F**) Comparisons of the percentages of C11-BODIPY^581/591^-positive CD14^+^ cells were evaluated among active AOSD patients, inactive AOSD patients, and HC subjects. The data are presented as box-plot diagrams, in which the box encompasses the 25th percentile (lower bar) to the 75th percentile (upper bar). The horizontal line within the box indicates the median value for each group. The *p*-values were determined by using the Kruskal–Wallis test. * *p*-value <  0.05, ** *p*-value <  0.01.

The results of previous studies showed that ferritin expression was higher in monocytes compared with other leucocytes [48,49]. Considering that the effects of ferritin on inflammatory responses mainly occur in CD14+ cells or monocytes/macrophages [29], we mainly investigated CD14+ cells among the PBMCs. The C11-BODIPY^581/591^ fluorescence intensity (MFI) that reflects LPO levels on PBMCs or CD14^+^ cells was quantified using flow cytometry analysis. The representative histograms of C11-BODIPY*^581/591^* fluorescence intensity on PBMCs (Figure 1A) or CD14^+^ cells (Figure 1C) were obtained from one active AOSD patient, one inactive AOSD patient, and one HC, respectively. A significantly higher MFI of LPO-expressing PBMCs was observed in active AOSD patients (4456, interquartile range (IQR) 4047–5307) compared with HC (1900, IQR 1015–3099, *p* < 0.05) (Figure 1B). Similarly, active AOSD patients had a significantly higher MFI on LPO-expressing CD14^+^-gated cells (median 9091, IQR 6193–10,200) than HC (4551, IQR 2085–6490, *p* < 0.01) (Figure 1D). As shown in Figure 1E, the percentage of LPO production was detected on CD14^+^ cells. The percentage of elevated LPO on CD14^+^ cells was significantly higher in active AOSD patients (median 97.96, IQR 90.0–99.5) than in HC subjects (median 87.3, IQR 60.1–87.5, *p* < 0.05), but no significant difference was observed between inactive AOSD patients (median 95.06, IQR 78.5–97.5) and HC subjects (Figure 1F).

### 3.3. LPO Inducers Increased the Expression of Ferritin and IL-1β on PBMCs from AOSD Patients

**Figure 2:**
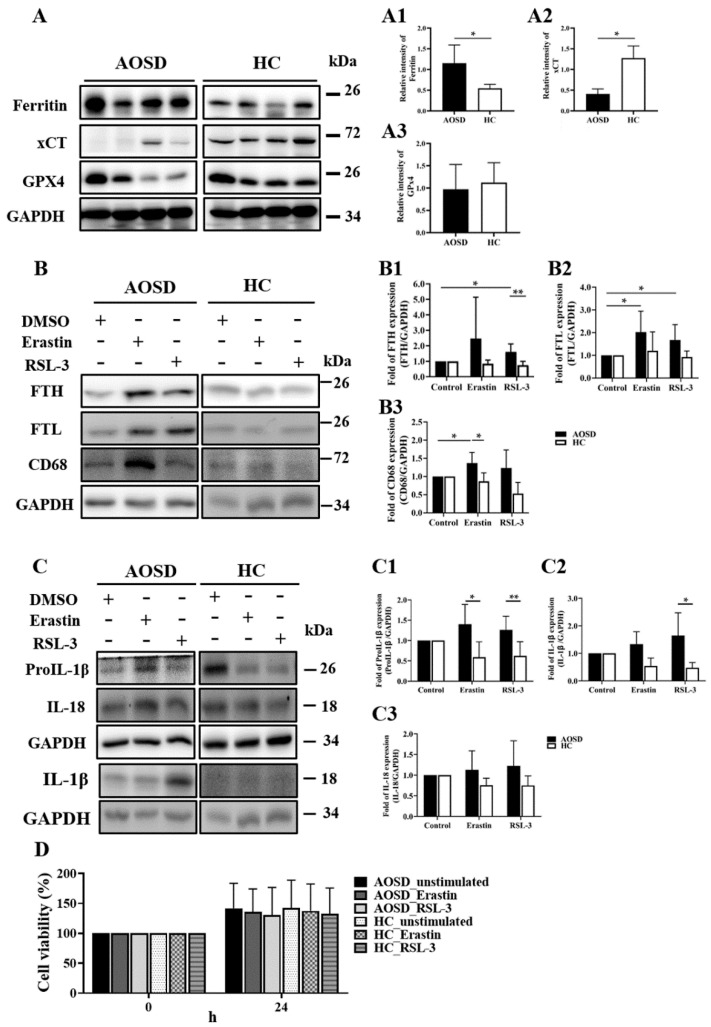
LPO inducers upregulated the expression of ferritin and IL-1β on PBMCs from AOSD patients. (**A**) The representative protein expression levels of ferritin, xCT, and GPX4 on PBMCs lysates obtained from AOSD patients (*n* = 4) and HC (*n* = 4). (**B**) Western blotting analysis results of the expression levels of (**B**) ferritin heavy chain (FTH), ferritin light chain (FTL), CD68 protein, (**C**) ProIL-1β, IL-1β, and IL-18 in the PBMCs from AOSD patients or HC subjects after incubation with ferroptosis inducers, erastin (20 μM) or RSL3 (10 μM), for 24 h. (**A1**–**A3**), (**B1**–**B3**) and (**C1**–**C3**) Proteins expression were evaluated by the Western blotting assay. The bars and error bars indicate mean and standard deviation, respectively. (**D**) After treatment with erastin (20 μM) or RSL3 (10 μM) for 24 h, the cell viability of PBMCs was measured by the WST-1 assay. PBMC: peripheral blood mononuclear cells; AOSD: adult-onset Still’s disease; HC: healthy controls; IL: interleukin; FTH: ferritin heavy chain; FTL: ferritin light chain. Bars and error bars indicate mean and standard deviation, respectively. The *p*-values were determined by the multiple comparison Bonferroni post-test. * *p*-value <  0.05, ** *p*-value <  0.01.

Similar to the elevated levels of serum ferritin, our AOSD patients had significantly higher expression levels of intracellular ferritin on PBMCs (median 1.02, IQR 0.82–1.61) compared with HC subjects (median 0.55, IQR 0.45–0.63, *p* < 0.05, Figure 2A). Given that both xCT and GPX4 are endogenous antioxidants acting against free iron-mediated LPO [37,38], the levels of xCT protein on PBMCs were significantly lower in AOSD patients (median 0.938, IQR 0.31–0.53) than in HC subjects (median 1.18, IQR 1.06–1.57, *p* < 0.05), but the expression levels of GPX4 were not significantly different between the two groups.

After stimulation of PBMCs with LPO inducers, erastin or RSL3, the FTH levels on the erastin-treated or RSL3-treated PBMCs from AOSD patients (mean of fold change, 2.47 and 1.61, respectively) were significantly higher than those from HC subjects (0.84, *p* < 0.05, and 0.74, *p* < 0.01, respectively, Figure 2B). The expression levels of FTL were also significantly enhanced on the erastin-treated and RSL3-treated PBMCs from AOSD patients, which was not observed in those from HC subjects. The CD68 is commonly used as a cytochemical and an active marker of monocytes/macrophages [50]. Hu et al. demonstrated that upregulation of CD68+ expression in macrophages could be induced by neutrophil extracellular traps from AOSD patients [51]. Besides, Ruscitti et al. revealed a positive correlation between FTH levels and the number of infiltrating FTH/CD68+ macrophages in the cutaneous lesions of AOSD patients [52]. Given the high CD68 expression in monocytes/macrophages, we examined the effect of LPO inducers on the CD68 expression on PBMCs. The CD68 expression levels on erastin-treated PBMCs from AOSD patients (mean of fold change, 1.37) were also significantly higher than those from HC subjects (0.86, *p* < 0.05) (Figure 2B).

Considering that lipopolysaccharide induced pro-IL-1β protein expression on PBMCs through the oxidation pathway [53], we examined the effect of LPO inducers on pro-inflammatory cytokine production. After stimulation of PBMCs with erastin or RSL3, pro-IL-1β protein expression levels were significantly higher in AOSD patients (mean of fold change, 1.40 and 1.26, respectively) compared with HC subjects (0.59, *p* < 0.05, and 0.62, *p* < 0.01, respectively) (Figure 2C). Upon RSL3 stimulation, IL-1β protein expression levels were also significantly increased in PBMC (mean of fold change, 1.62) from AOSD patients compared to those of HC subjects (mean of fold change, 0.42, *p* < 0.05) (Figure 2C). However, there was no significant difference in IL-18 expression levels on erastin-treated or RSL3-treated PBMCs between AOSD patients and HC subjects (Figure 2C).

Erastin and RSL3 are not only LPO inducers but also ferroptosis inducers that may affect cell viability. However, in the present study, we revealed no significant change in the cell viability of PBMCs treated with erastin or RSL3 between AOSD patients and HC (Figure 2D).

### 3.4. LPO Inducers Increased the Expression Levels of LPO, FTH, and FTL on Monocytes or Macrophages from AOSD Patients and HC Subjects

**Figure 3:**
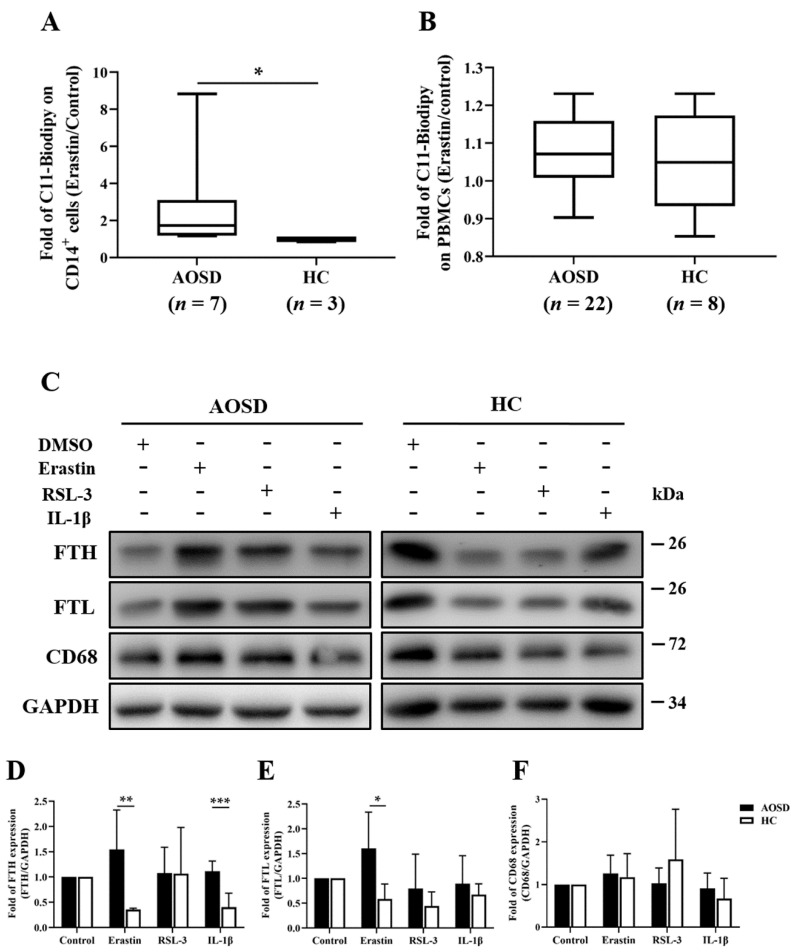
LPO inducers increased the expression levels of LPO, FTH, and FTL on monocytes/macrophages from AOSD patients and HC subjects. After incubation of CD14^+^ monocytes (**A**) and PBMCs (**B**) with erastin (20 μM) for 24 h, the C11-BODIPY^581/591^ fluorescence intensity on circulating CD14^+^-gated cells or PBMC-gated cells was determined by flow cytometry. Fold change of C11-BODIPY^581/591^ MFI was calculated as the LPO MFI on erastin-treated cells/those on untreated cells. Data are presented as box-plot diagrams, with the box encompassing the 25th percentile (lower bar) to the 75th percentile (upper bar). The horizontal line within the box indicates the median value respectively for each group. The *p*-values were determined using the nonparametric Mann–Whitney U test. * *p*-value < 0.05, ** *p*-value < 0.01. (**C**) After the macrophages isolated from AOSD patients or HC subjects were stimulated with erastin (20 μM), RSL3 (10 μM), or IL-1β (2 ng/mL) separately for 24 h, the levels of (**D**) FTH, (**E**) FTL, and (**F**) CD68 expression were evaluated by the Western blotting assay. The bars and error bars indicate mean and standard deviation, respectively. The *p*-values were determined by the multiple comparison Bonferroni post-tests. * *p*-value < 0.05, ** *p*-value < 0.01, and *** *p*-value < 0.001.

Compared with HC subjects, active AOSD patients had significantly higher LPO levels on erastin-treated CD14^+^ cells (median fold, 1.71, IQR 1.19–3.10 versus 0.97, IQR 0.84–1.07, *p* < 0.05) (Figure 3A), but this was not observed on erastin-treated PBMCs (Figure 3B). Subsequently, we obtained monocyte-derived macrophages by using the stimulation of peripheral blood CD14^+^ cells with GM-CSF, and examined the expression levels of FTH, FTL, and CD68 on macrophages after treatment with erastin, RSL3, or IL-β. The results showed significantly higher FTH levels on erastin-treated and IL-1β-treated macrophages from AOSD patients (mean fold, 1.54 and 1.13, respectively) compared with those from HC subjects (0.35 and 0.40 respectively, both *p* < 0.001, Figure 3C,D). Similarly, the FTL levels were significantly higher on the erastin-treated macrophages from AOSD patients (mean fold, 1.60) than from HC subjects (0.35, *p* < 0.05, Figure 3E). However, there was no significant difference in CD68 expression levels on macrophages between AOSD patients and HC subjects (Figure 3C,F).

### 3.5. Plasma Levels of TBARS (MDA) and 4-HNE and Their Relation with Inflammatory Parameters or Cytokines in AOSD Patients 

**Figure 4:**
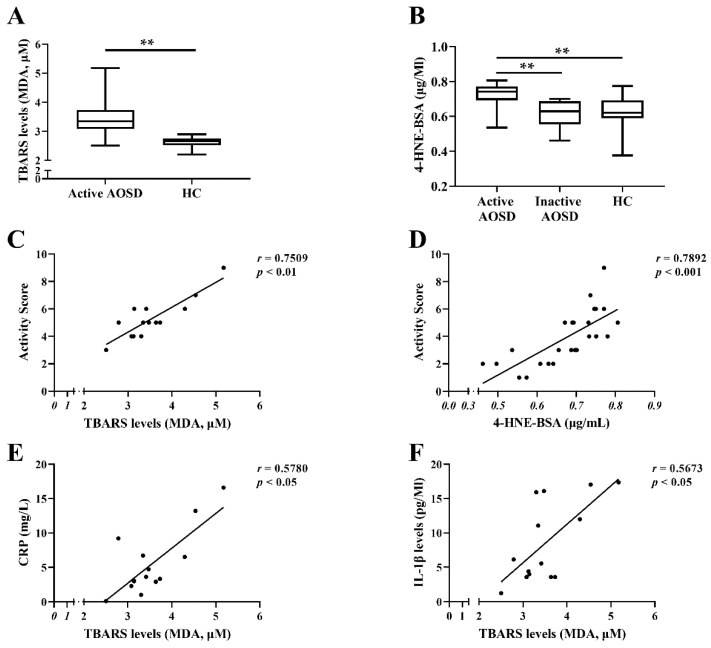

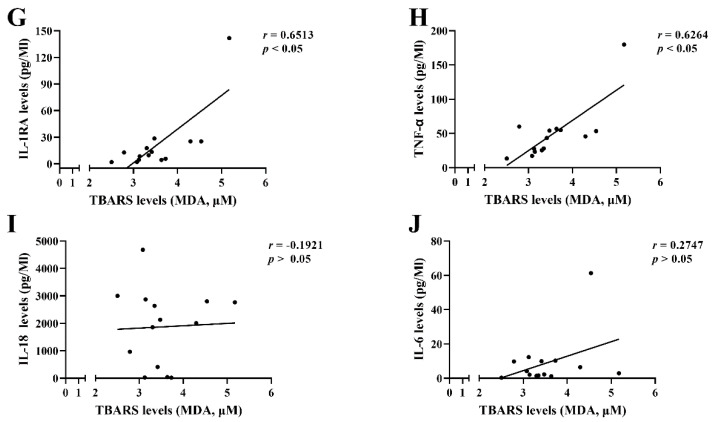
Plasma levels of LPO-related metabolites and their relation to disease activity and inflammatory parameters in AOSD patients. (**A**) Comparison of plasma MDA (malondialdehyde) levels between active AOSD patients (*n* = 14) and HC subjects (*n* = 9). (**B**) Comparison of plasma 4-hydroxyalkenals (4-HNE) levels among active AOSD patients (*n* = 14), inactive AOSD patients (*n* = 11), and HC subjects (*n* = 9). The correlation of plasma levels of (**C**) MDA or (**D**) 4-HNE with disease activity scores, or between MDA levels and (**E**) CRP, (**F**) IL-1β, (**G**) IL-1RA, (**H**) TNF-α, (**I**) IL-18, or (**J**) IL-6. The *p*-values were determined by using the Kruskal–Wallis test. ** *p*-value <  0.01. The correlation of 4-HNE-BSA levels with activity scores (**D**). The correlation of MDA levels with (**C**) activity scores, (**E**) CRP, (**F**) IL-1β, (**G**) IL-1RA, (**H**) TNF-α, (**I**) IL-18, or (**J**) IL-6. The *p*-values were determined by Spearman’s test. AOSD: adult-onset Still’s disease; CRP: C-reactive protein; HC: healthy controls; IL: interleukin; IL-1RA: interleukin-1 receptor antagonist; TNF: tumor necrosis factor.

Given that TBARS (MDA) and 4-HNE are potential biomarkers of LPO [39], we also evaluated the relation between plasma levels of TBARS (MDA) or 4-HNE and inflammatory parameters in AOSD. The MDA, the end product of polyunsaturated fatty acids’ peroxidation, could react with thiobarbituric acid (TBA) to form MDA-TBA2 adducts, also called the thiobarbituric acid reactive substance (TBARS). With maximum absorbance at 532 nm and fluorescence Excitation/Emission at 532/553 nm, the TBARS assay was used to measure one of the lipid peroxidation products, MDA [54]. As shown in Figure 4A,B, active AOSD patients had significantly higher levels of plasma TBARS (MDA) and 4-HNE-BSA (median 3.34, IQR 3.07–3.73, and 0.74, IQR 0.69–0.77, respectively) than HC subjects (median 2.65, IQR 2.51–2.75, *p* < 0.01, and 0.62, IQR 0.59–0.69, *p* < 0.01, respectively). Besides, plasma 4-HNE-BSA levels were significantly increased in active AOSD patients compared to inactive AOSD patients (median 0.62, IQR 0.55–0.69, *p* < 0.01) (Figure 4B). In AOSD patients, plasma levels of TBARS (MDA) or 4-HNE-BSA were positively correlated with systemic activity scores (Figure 4C,D). Plasma TBARS (MDA) levels were also significantly correlated with the levels of C-reactive protein (CRP) or pro-inflammatory cytokines, including IL-1β, IL-1RA, and TNF-α, in active AOSD patients (Figure 4E–H). However, there was no significant correlation between plasma TBARS (MDA) levels and plasma levels of IL-18 or IL-6 (Figure 4I,J).

## 4. Discussion

Lipid peroxidation (LPO) is crucial for the pathogenesis of inflammatory diseases [35]. However, the relation between LPO and inflammatory parameters in AOSD, including ferritin levels, remains unexplored. This study is the first that uses flow cytometry analysis to demonstrate significantly higher LPO levels in circulating monocytes and PBMCs of AOSD patients compared with HC subjects. Consistent with two previous studies [2,21], our AOSD patients showed significantly higher ferritin expression levels on PBMCs than HC subjects. To explore the link between LPO and ferritin expression, we further evaluated the ferritin expression levels on PBMCs and macrophages treated with LPO inducers erastin or RSL3. The results showed that both erastin and RSL3 could significantly enhance ferritin expressions, including FTH and FTL, and the increment was greater in AOSD patients than in HC subjects, suggesting an association between increased LPO stress and elevated ferritin in AOSD. Besides, the levels of TBARS (MDA) and 4-HNE, the LPO downstream metabolites, were significantly increased and correlated with systemic disease activity scores in our AOSD patients. These findings indicate the potential involvement of LPO stress in AOSD pathogenesis.

AOSD patients have been reported to have high circulating levels of ferritin [2,21]. Ferritin, the second-largest intracellular iron pool, plays an important role in the pathogenesis of inflammation [22,29]. Resonating with these observations, our AOSD patients had significantly higher expression levels of intracellular ferritin in PBMCs than HC subjects. Regarding the immunomodulatory role of ferritin, certain acidic isoferritins homologous to FTH, such as placental 43 kda isoferritin (p43-PLF), are involved in the immunosuppressive activity in pregnancy [55]. Bresgen et al. also revealed that ferritin could contribute to apoptosis in primary hepatocytes through Fas stimulation and proapoptotic mitochondrial signaling [56,57].

Ferroptosis has recently been regarded as an iron-dependent form of non-apoptotic cell death [36]. There is a need for a fine-tuned mechanism to maintain iron homeostasis that may fulfill the need for iron but avoid the toxicity caused by an excess of ROS [36,37]. Cysteine acts as a substrate for glutathione biosynthesis, which is further used to activate GPX4 to inhibit lipid peroxidation. Intracellular cysteine levels are mainly regulated by system Xc, also known as system xCT, which is a cystine/glutamate antiporter. Therefore, xCT is considered as an endogenous antioxidant for free-iron-mediated LPO [37,38]. We similarly revealed significantly lower xCT protein levels in PBMCs in the AOSD patients compared with HC subjects, and accordingly, significantly higher LPO levels on circulating PBMCs and monocytes in the AOSD patients compared with HC subjects.

Although ferritin levels’ elevation is a feature of AOSD [2,21], the relation between ferritin expression and LPO in AOSD has yet to be explored. Given that erastin and RSL3 are ferroptosis inducers capable of inducing lipid oxidation, we examined the effects of both inducers on the expression of ferritin subunits FTH and FTL in PBMCs and macrophages. After stimulation with erastin or RSL3, there was an upregulation of ferritin expression levels in PBMCs of the AOSD patients, and the increments were significantly greater compared with HC subjects. Similarly, the ferritin expression on erastin- or RSL3-treated macrophages from the AOSD patients was significantly higher than that from HC subjects. The results indicate that ferritin expression could be regulated by oxidative stress [58]. Besides, both LPO inducers could enhance IL-1β expression, and the increments were greater in the AOSD patients than in HC subjects, suggesting a potential effect of LPO on the production of proinflammatory cytokines [59]. As ferritin synthesis can also be upregulated by inflammatory cytokines [60], the ferritin expression on macrophages from our AOSD patients was further enhanced by IL-1β. These observations suggest a link between increased LPO and elevated ferritin expression in AOSD, at least partly through IL-1β augmentation. However, the causal relationship needs further clarification in future studies.

Ruscitti et al. demonstrated an increased number of CD68(+)/H-ferritin (+) cells on macrophages in lymph nodes from active AOSD patients [61]. In the present study, we revealed that LPO inducers could upregulate the expression levels of both CD68 and ferritin in PBMCs from AOSD patients, suggesting a pathogenic role of LPO in the expression of ferritin in this inflammatory disease. Besides, Ruscitti et al. demonstrated that exogenous FTH stimulation could regulate NLRP3 expression and IL-1β production on macrophages [29]. We revealed that IL-1β could also upregulate FTH expression on macrophages or PBMCs from AOSD patients. Since LPO oxidation stress can induce FTH expression and modulate inflammation response, we speculate that LPO stress is a potential modulator of intracellular iron homeostasis in the inflammatory diseases.

Given that erastin and RSL-3 are also the inducers of ferroptosis, both inducers may affect cell viability [36]. However, we revealed no significant differences in the cell viability of PBMCs treated with erastin or RSL3 between AOSD and HC. Similar to our findings, Wang et al. reported that erastin could even promote PBMCs’ proliferation and differentiation through induction of LPO [62], indicating that LPO could initiate ferroptotic signaling, but not necessarily induce cell death.

Elevated circulating TBARS (MDA) levels have been found in patients with autoimmune diseases such as systemic lupus erythematosus (SLE) and rheumatoid arthritis (RA) [63,64]. Yin et al. also revealed that lipid peroxidation by 4-HNE treatment may indeed induce inflammation reactions in synoviocytes in RA [65]. We similarly demonstrated that AOSD patients had elevated levels of TBARS (MDA) and 4-HNE, which were positively correlated with disease activity scores. These results suggest that TBARS (MDA) or 4-HNE may be an activity indicator in AOSD patients. Similar to the findings that a significant correlation between TBARS (MDA) and TNF-α levels was found in severe dengue cases [66], a positive correlation between TBARS (MDA) levels and proinflammatory cytokines, including L-1β and TNF-α, was observed in our patients. Besides, our results showed that plasma TBARS (MDA) levels were positively correlated with levels of IL-1Ra, an inhibitory cytokine that controls inflammatory responses and plays a compensatory role in fine-tuning the immune response.

Despite the novel findings, there are some limitations of our study. This preliminary pilot study enrolled a limited number of active AOSD patients. The lack of a significant correlation between FTH and TBARS (MDA) levels in AOSD patients might be due to the small sample size. In addition, we did not elucidate the effect of lipid oxidation/its metabolites on NLRP3 inflammasome signaling in AOSD patients because the sample size was not large enough to be tested. Therefore, future studies dissecting the biological role of lipid peroxidation in the FTH-NLRP3 inflammasome signaling axis in macrophages are certainly needed.

## 5. Conclusions

For the first time, we demonstrated elevated LPO levels in circulating PBMCs and monocytes and augmented expression levels of ferritin and IL-1β in response to LPO inducers in AOSD patients but not in HC subjects. The plasma levels of TBARS (MDA) and 4-HNE, the LPO downstream metabolites, were increased and positively correlated with the disease activity score in active AOSD patients. The TBARS (MDA) levels were also significantly correlated with inflammatory parameters and cytokines in AOSD patients. These findings suggest that LPO may be linked to elevated ferritin levels and participate in AOSD pathogenesis.

## Data Availability

The datasets used and/or analyzed during the current study are available from the corresponding author upon reasonable request.
